# Artificial rearing influences the morphology, permeability and redox state of the gastrointestinal tract of low and normal birth weight piglets

**DOI:** 10.1186/s40104-017-0159-3

**Published:** 2017-04-08

**Authors:** Hans Vergauwen, Jeroen Degroote, Sara Prims, Wei Wang, Erik Fransen, Stefaan De Smet, Christophe Casteleyn, Steven Van Cruchten, Joris Michiels, Chris Van Ginneken

**Affiliations:** 1 Laboratory of Applied Veterinary Morphology, Department of Veterinary Sciences, Faculty of Biomedical, Pharmaceutical and Veterinary Sciences, University of Antwerp, Campus Drie Eiken, Universiteitsplein 1, D.U.015, 2610 Wilrijk, Belgium; 2 Department of Applied Biosciences, Faculty of Bioscience Engineering, Ghent University, Ghent, Belgium; 3 Laboratory for Animal Nutrition and Animal Product Quality (LANUPRO), Department of Animal Production, Faculty of Bioscience Engineering, Ghent University, Melle, Belgium; 4 StatUa Center for Statistics, University of Antwerp, Antwerp, Belgium

**Keywords:** Milk replacer, Oxidative stress, Small intestine, Suckling period, Tight junction proteins

## Abstract

**Background:**

In this study the physiological implications of artificial rearing were investigated. Low (LBW) and normal birth weight (NBW) piglets were compared as they might react differently to stressors caused by artificial rearing. In total, 42 pairs of LBW and NBW piglets from 16 litters suckled the sow until d19 of age or were artificially reared starting at d3 until d19 of age. Blood and tissue samples that were collected after euthanasia at 0, 3, 5, 8 and 19 d of age. Histology, ELISA, and Ussing chamber analysis were used to study proximal and distal small intestine histo-morphology, proliferation, apoptosis, tight junction protein expression, and permeability. Furthermore, small intestine, liver and systemic redox parameters (GSH, GSSG, GSH-Px and MDA) were investigated using HPLC.

**Results:**

LBW and NBW artificially reared piglets weighed respectively 40 and 33% more than LBW and NBW sow-reared piglets at d19 (*P* < 0.01). Transferring piglets to a nursery at d3 resulted in villus atrophy, increased intestinal FD-4 and HRP permeability and elevated GSSG/GSH ratio in the distal small intestine at d5 (*P* < 0.05). GSH concentrations in the proximal small intestine remained stable, while they decreased in the liver (*P* < 0.05). From d5 until d19, villus width and crypt depth increased, whereas PCNA, caspase-3, occludin and claudin-3 protein expressions were reduced. GSH, GSSG and permeability recovered in artificially reared piglets (*P* < 0.05).

**Conclusion:**

The results suggest that artificial rearing altered the morphology, permeability and redox state without compromising piglet performance. The observed effects were not depending on birth weight.

## Background

The neonatal period in the pig’s life is accompanied with high morbidity and mortality [1, 2]. In addition, increasing litter sizes in modern swine production have led to higher rates of piglets born with a low birth weight (LBW) [3]. Both newborn LBW human [4–6] and LBW piglets [7–10] seem to have a lower capacity to mount an antioxidant response. Newborns transitioning from maternal mediated respiration to autonomous pulmonary respiration outside the uterus are suddenly exposed to O_2_-derived free radicals [11]. This increased the production of reactive species in various organs [7]. A redox imbalance affects cellular signaling, protein synthesis, and enhances proteolysis that can ultimately lead to a dysfunctional intestinal barrier and suboptimal regenerative potential as shown in vitro [12–15]. Consequently, the observed redox imbalance and the downstream effects could explain the abnormal absorption and metabolism of nutrients, reduced growth and impaired development of the small intestine, liver, and muscle observed in LBW piglets [10, 16–21]. This redox imbalance appears to persist beyond weaning [9]. Wang et al. observed that mRNA expression of occludin, heme oxygenase 1, catalase, thioredoxin reductase genes and occludin protein expression continued to be lower in LBW pigs during the suckling period [21]. This apparently conflicts with the observation that LBW piglets that survive the critical first days after birth show an intestinal morphology, digestive capacity, cytokine production, intestinal motility and permeability that is comparable with those seen in normal birth weight (NBW) littermates [22–24].

The increasing incidence of supernumerary and LBW piglets has raised an urgent need for innovative rearing strategies [1]. Next to cross-fostering [25], supplementing piglets [26] and split nursing [27], piglets can be transferred to a nursery and artificially reared [28–30]. Similar to conventional weaning, the artificially reared piglet encounters psychological and physical stressors including maternal and littermate separation, abrupt changes in diet composition and environment, lower intake of bioactive substances, as well as unfamiliar drinking nipples, increased exposure to pathogens and antigens, comingling and establishment of social hierarchy with unfamiliar pigs from different litters. The physiological responses to the strategy of full artificial rearing are largely unknown. Conventional weaning is associated with the induction of intestinal oxidative stress [31–33] and LBW piglets have more difficulties to maintain a balanced redox state when exposed to weaning stressors [9]. It is unknown at present if the response to artificial rearing, since it includes similar stressors as conventional weaning, is different in LBW piglets—which have a lower antioxidant capacity [21]—compared to NBW piglets.

Therefore, we aimed to investigate the impact of artificial rearing on piglet performance, proximal and distal small intestinal (SI) morphology, mitosis, apoptosis, and tight junction protein expression, permeability, and SI, liver and systemic redox state development compared to conventional rearing. Given the similarities between conventional weaning stressors and artificial rearing stressors we hypothesized that artificial rearing results in a redox imbalance and negatively affects intestinal morphology and functionality. Secondly, given the differences observed between NBW and LBW piglets during the suckling period, we hypothesized that in view of their affected redox state, the morphology and functionality of the small intestine is suboptimal in LBW piglets and that artificial rearing has a greater negative impact in this birth weight category.

## Methods

### Pig model and tissue collection

Eighty-four piglets (Topigs hybrid × Piétrain) were selected during two consecutive farrowing rounds. Only sows with 14 or more live-born piglets were selected and no cross-fostered piglets were included in the experiment. Piglets were tagged and weighed within 12 h after parturition. A LBW piglet was defined as a pig having a birth weight between 0.75 and 0.90 kg and belonging to the lower quartile of litter birth weights. A NBW piglet had a birth weight within 1 SD unit of the mean birth weight of the whole litter. The average birth weight of all the piglets used in this study was 0.81 ± 0.01 kg and 1.26 ± 0.02 kg for LBW and NBW piglets, respectively. Forty-two pairs of LBW and NBW gender-matched littermates were either left to suckle the sow or transferred to a nursery at the age of 3 d and had ad libitum access to a milk replacer that was refreshed twice a day (Table 1). Milk replacer was made by mixing 200 g of milk replacer powder into 1 L of water, preheated at 42 °C. This resulted in a dry matter (DM) content of 193 g/kg milk. This is comparable with the DM content of sow milk, which is around 170 to 190 g/kg milk between 3 d and 42 d of lactation [34]. In Table 1, the calculated nutrient composition of some important nutrients is shown, expressed on DM basis. When account for the dilution factor, it is possible to compare this milk formula with sow milk. For instance, it is clear that the crude protein level in the formula (49.8 g/kg milk) is low but comparable with what can be expected in sow milk (51–64 g/kg milk) ([34–36]). The crude fat content was calculated at 22 g/kg milk, which is considerably lower than the crude fat content of sow milk (5.3–6.5 g/kg milk) [34–36]. In contrast, the total amount of lactose in the liquid milk replacer was 80 g/kg milk. Values for sow milk range from 48 to 59 g/kg milk, and thus are lower than what was offered to the formula fed piglets. From this, it might be clear that the milk formula was not completely comparable to the nutrient composition of porcine milk. However, this tailor made milk formula was meant as a compromise of many existing, commercially available milk replacer formulas, and as a copy of sow milk as such. A maximum of 4 NBW-LBW gender matched littermate pairs were co-housed. The starting ambient temperature was 32 °C and linearly decreased to 28 °C towards d19 post-natal. Heating and ventilation was automatically controlled in function of the temperature settings. Six pairs of sow-reared LBW and NBW piglets were sampled on d0, d3, d8 and d19 of age, and 6 pairs of artificially reared LBW and NBW piglets were sampled on d5, d8 and d19 of age. Piglets sampled on d0 were removed from the sow between 12 and 24 h after parturition. At d3, intramuscular iron injections were given (Iron(III) Dextran, 200 mg/piglet, Uniferon). The selected animals did not receive any antibiotic treatment prior or during the experiment.

**Table 1:** Composition of the milk replacer used for piglets from 3 d of age until weaning at d 19

Ingredient composition, %	
Coco fat filled whey 50/50	42.00
Skimmed milk powder	17.61
Whey permeate	8.29
Soy protein concentrate Soycomil K	10.00
Cheddar whey powder	8.29
Whey protein concentrate80, DVN	7.00
Spray dried blood plasma P80	4.00
Dicalciumphosphate 18% P	0.32
DL-Methionine	0.31
Citric acid	0.30
L-Tryptophan	0.08
Vitamin and mineral premix^a^	1.8
Calculated nutrient levels	
NEv(1997) MJ/kg	15.48
CP, g/kg	249
CF, g/kg	110
dLYS, g/kg	18.2
dMET + CYS, g/kg	11.5
dTHR, g/kg	11.2
dTRY, g/kg	4.1

^a^The mineral and vitamin premix supplied as the following (per kg diet): Vitamin A, 30, 000 IU; Vitamin D_3_, 5000 IU; Vitamin E; 75 mg; Fe^2+^, 120 mg; Zn^2+^, 35 mg; Cu^2+^, 135 mg;Mn^2+^, 45 mg; Se^6+^, 350 μg; I^-^, 1 mg, BHT, 75 mg/kg

Pigs were killed by exsanguination by severing the carotid arteries and jugular veins following induction of terminal anesthesia by intramuscular injection of ketamine (15 mg/kg BW) combined with xylazine (2 mg/kg BW). All piglets were weighed prior to euthanasia.

Blood was collected in EDTA and heparinized tubes containing supplemental bathophenanthroline disulfonate sodium salt. Erythrocytes were isolated by centrifuging (3000 × g, 15 min) 0.5 mL of unclotted, heparinized blood. After removing the plasma, erythrocytes were lysed by adding 100 μL of a 70% metaphosphoric acid solution, 600 μL milli Q and intense vortexing. These extracts were then centrifuged (3000 × g, 15 min), and 0.5 mL of the remaining acid extract was transferred to a vial containing 50 μL of a γ-glu-glu internal standard solution. After opening the abdomen, the liver was isolated and samples of the left lateral lobe were dissected for acid and phosphate buffered aqueous extraction, as described for the small intestinal mucosa. Subsequently, the small intestine (SI), defined as the part of the gastrointestinal tract between the pylorus and the ileocecal valve, was dissected and its length was measured. A 10 cm segment of proximal and distal SI (5 and 75% of total SI length, respectively) was taken for Ussing chamber measurements. In addition, 20 cm segments at 5 and 75% of the total SI length were emptied and carefully flushed with saline. The tissue of these 20 cm segments was placed on an ice-cold surface and the mucosa was retrieved by gently scraping the mucosal surface with a glass slide. Aliquots of the mucosa were either used instantaneously for acid and phosphate buffered aqueous extracts or transferred to plastic 2 mL screw-capped tubes, snap-frozen in liquid nitrogen and stored at -80 °C pending redox state analysis. Furthermore, 5 cm segments at 5 or 75% of the total SI length were taken, flushed with saline, snap-frozen in liquid nitrogen and stored at -80 °C pending protein expression analysis. Finally, a 5 cm segment at 5 or 75% of the total SI length was flushed with saline, divided in smaller pieces of max 1.5 cm in length and fixated for 2 h in 4% freshly prepared paraformaldehyde (in 0.01 mol/L phosphate-buffered saline) (volume tissue/volume fixative: 1/5) and routinely processed for paraffin-embedding [37].

### Small intestinal histo-morphological measurements

In brief, 4 μm sections of paraffin-embedded samples were mounted on slides and stained with hematoxylin-eosin. Villus height, mid-villus width, and crypt depth were measured at 10× magnification using an Olympus BX61 microscope and image analysis software (analySIS Pro, Olympus Belgium, Aartselaar, Belgium) in 1–3 well-oriented villi and associated crypts in at least 12–15 sections per tissue sample, to yield 30 measurements per small intestinal region.

### Small intestinal protein expression profile analysis

The concentration of specific tight junction proteins and markers for apoptosis and mitosis of the proximal and distal intestinal tissue samples was investigated using commercially available enzyme-linked immunosorbent assays (ELISA) of occludin (SEC228Hu), claudin-3 (SEF293Hu), proliferating cell nuclear antigen (PCNA) (SEA591Hu) and caspase-3 (SEA626Hu) (Cloud-Clone Corporation®, Houston, TX, USA). All tissue samples were crushed, dissolved in phosphate-buffered saline solution (PBS, pH 7.4, 0.01 mol/L), sonicated 6 times for 5 s at 4 °C (Sonics Vibracell™, VCX130, Newtown CT, USA), and kept on ice for 30 min. Subsequently the samples were centrifuged for 2 min at 13,400 rpm at 4 °C (Heraeus X3R with TX-750, Thermo Scientific, Rockford, USA), after which the supernatant was isolated, total protein concentration was determined using a Pierce TM BCA Protein Assay Kit (Thermo Scientific, Rockford, USA) and finally the samples were diluted to a total protein concentration of 10 ng/μL. Then, samples were processed on a sandwich ELISA plate and the experiment was performed according to the manufacturer’s instructions. Absorbance was measured using an Infinite M200 Pro spectrophotometer with X-Fluor software at 450 nm at 25 °C (Tecan Group Ltd., Männedorf, Switzerland). Values of protein expression were determined per gram of total protein in a sample, measured using a Pierce^TM^ BCA Protein Assay Kit (ThermoFisher Scientific, Belgium), and expressed as fmol/mg.

### Ex vivo measurement of small intestinal permeability

Intestinal mucosal permeability was assessed ex vivo by measuring the translocation of macromolecular markers using the Ussing chamber technique. The segments were first rinsed with saline. The mucosal layer was stripped from the seromuscular layer and pinned onto 1.07 cm^2^ sliders that were mounted into modified Ussing chambers (Dipl.-Ing. Muβler Scientific Instruments, Aachen, Germany). All tissues were mounted within 10 min following euthanasia. Tissues were immersed in 6.5 mL Ringer solution (115 mmol/L NaCl, 5 mmol/L KCl, 25 mmol/L NaHCO_3_, 2.4 mmol/L Na_2_HPO_4_, 0.4 mmol/L NaH_2_PO_4_, 1.25 mmol/L CaCl_2_, 1 mmol/L MgSO_4_) with 6 mmol/L of mannitol or glucose in the luminal and serosal side, respectively. The system was water-jacketed to 37 °C and oxygenated with 95% O_2_ and 5% CO_2_. After an equilibration period of 20 min, 4 kDa fluorescein isothiocyanate-dextran (FD-4, Sigma-Aldrich, Bornem, Belgium) and 40 kDa horseradish peroxidase (HRP, type IV, Sigma-Aldrich, Bornem, Belgium) were added to the mucosal side to a final concentration of 0.8 mg/mL of FD-4 and 0.4 mg/mL of HRP. Samples of the buffer solution were taken from the serosal chamber at 20, 40, 60 and 100 min after adding markers. Meanwhile, the same volume of buffer was taken from the mucosal side to keep the volume balance across sides. Fluorescence intensity of FD-4 in the medium was measured at excitation wavelength of 485 nm and emission wavelength of 538 nm using a fluorescence plate reader (Thermo Scientific, Marietta, OH, USA). HRP was measured according to the method described previously [38]. In short, HRP activity was measured by adding a ‘start solution’ (50 mL of 0.2 mol/L NaH_2_PO_4_, 1 mL of 0.2 mol/L Na_2_HPO_4_, 20.4 μL of 30% H_2_O_2_, 1.7 mL of 1% dianisidine peroxide substrate, made up to 204 mL with water) to the HRP samples and left for 10 min, after which the reaction was terminated by addition of 120 μL of 4% sodium azide and the absorbance was read at 460 nm. The relation between peroxidase concentration and absorbance is linear in the concentration range 10–100 pmol/L. The apparent permeation coefficient (Papp) was calculated as:

$$ \mathrm{Papp}\ \left(\mathrm{cm}/\mathrm{s}\right):\ \left(\mathrm{dc}/\mathrm{dt}\right) \times \mathrm{V}/{\mathrm{c}}_0/\mathrm{A}, $$

Whereby dc/dt is the change of serosal concentration in the 20- to 100-min period (cm/s); V is the volume of the chamber, c_0_ is the initial marker concentration in the mucosal reservoirs and A the area of the exposed intestine in the chambers (cm^2^).

### Mucosal, liver and blood homogenate extracts and biochemical assays

An acid extract was prepared from 1 g of homogenized (Braun homogenizer at 900 rpm) intestinal mucosa or liver that was placed in 10 mL ice-cold perchloric acid (PCA) 10% solution and centrifuged at 15,000 × g for 15 min at 4 °C. The resulting acid extract (0.5 mL) was transferred to tubes containing 50 μL γ-glu-glu internal standard solution. Samples were snap frozen in liquid nitrogen and stored at -80 °C until analysis of GSH and glutathione disulfide (GSSG). The biuret reaction was applied to determine the total protein content. Mucosal GSH and GSSG were measured using a modified high performance liquid chromatography (HPLC) method [39, 40]. The derivation procedure included the reaction of 100 mmol/L iodoacetic acid solution with thiols to form S-carboxymethyl derivatives followed by chromophore derivation of primary amines with dinitrofluorobenzene (DNFB, 1% (v/v) in ethanol). GSH and GSSG were separated through EC250/4.6 Nucleosil 120-7 NH_2_ aminopropyl column (Machery-Nagel, Düren, Germany) protected by the same NH_2_ guard column (CC8/4). Chromatographic runs were performed at a flow-rate of 1.5 mL/min, starting at 80% solvent A/20% solvent B for 5 min followed by a 10 min linear gradient to 1% solvent A/99% solvent B and a 10 min isocratic period at 1% solvent A/99% solvent B (solvent A: water-methanol solution (1:4, v/v), solvent B: 0.5 mol/L sodium acetate–64% methanol). The column was re-equilibrated to the initial conditions for 15 min while maintaining the column temperature at 40 °C. The UV detector was set at 365 nm for absorption measurements. GSH and GSSG were identified by retention times of authentic standards. Concentrations were determined by using the internal and external standards and expressed as μmol/g protein. In addition, a phosphate buffered aqueous extract was made by mixing approximately 1 g of homogenized mucosa in 10 mL ice cold 1% Triton-X-100 phosphate buffer solution (pH = 7.0), by using an Ultra-Turrax dispensing machine (IKA-Werke GmbH & Co. KG, Staufen, Germany). The supernatant was transferred to 2 mL tubes, snap frozen and stored at -80 °C until analysis. Supernatants were used for the determination of GSH-Px activity and malondialdehyde (MDA; expressed as nmol/g protein) concentration. Assessment of GSH-Px activity (expressed as U/g protein) in EDTA plasma and mucosa was determined spectrophotometrically [41]. The thiobarbituric acid reactive substances (TBARS) method was used to measure MDA concentration in EDTA plasma, liver and mucosa extracts [42].

### Statistical analysis

Linear mixed models were fitted to assess the influence of birth weight category (NBW/LBW), feeding (artificially reared/sow-reared) and days postnatal (as a categorical variable) on the quantitative outcome variables. To model the dependence between observations within the same litter, random intercept terms for litter were added to the model. Depending on the research question, separate analyses were carried out in subgroups (e.g. only in sow-reared piglets) or time points were analyzed separately. In the subgroup analyses where no random effect terms was needed, a multiple linear regression model was fitted. Post hoc tests to compare mean values between the different time points (days postnatal) were carried out using Tukey’s honestly significant difference. Models were fitted using the Mixed Model procedure of the JMP Pro11 software (SAS Institute, Cary, NC, USA). Significance for the fixed effects was tested using an F-test with Kenward-Roger correction. Data are expressed as means and their standard errors (S.E.), and *P <* 0.05 was considered significant.

## Results

### Body weight of piglets

**Fig. 1:**
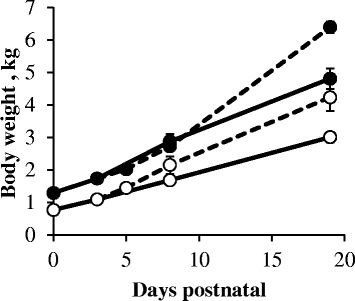
Body weights (kg) of LBW (open circle) and NBW (*closed circle*) from sow- (*full line*) and artificially reared (*dashed line*) piglets during the suckling period. Values are means ± SE (*n* = 6)

Average body weight of LBW and NBW piglets sampled at birth (d0) was 0.77 ± 0.07 and 1.29 ± 0.08 kg, respectively (Fig. 1). The body weight of both birth weight categories did not significantly change during the first 3 days. Afterwards, in both sow- and artificially reared piglets the body weight gradually increased (*P* < 0.001). At each point in time, LBW sow-reared piglets showed lower body weights compared to their NBW sow-reared littermates (*P* < 0.01). A similar observation was seen in artificially reared piglets at d5 (*P* < 0.001) and d19 (*P* < 0.05). Body weights of LBW and NBW artificially reared piglets were significantly higher, compared to respectively LBW and NBW sow-reared piglets at d19 (*P* < 0.05).

### Histo-morphological measurements in the proximal and distal small intestine

**Fig. 2:**
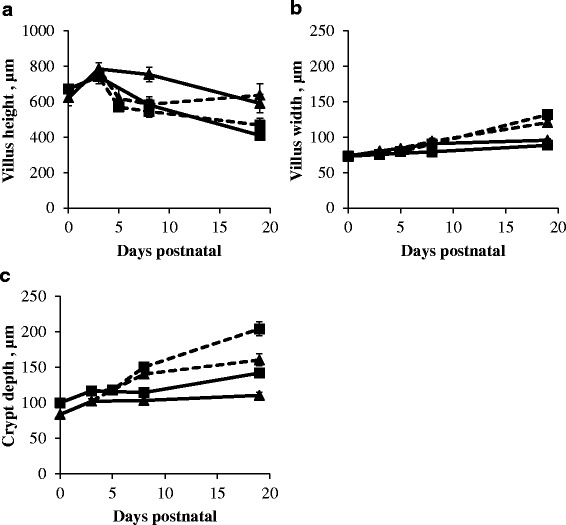
Villus height (μm) (**a**), villus width (μm) (**b**) and crypt depth (μm) (**c**) in proximal (*square*) and distal (*triangle*) SI of sow- (*full line*) and artificially reared (*dashed line*) piglets expressed as μm. Values are means ± SE (*n* = 12 as LBW and NBW piglets were pooled together)

The histo-morphological parameters were stable between d0 and d3 (Fig. 2). After d3, villus height significantly decreased (*P* < 0.05; Fig. 2a), whereas villus width (*P* < 0.05; Fig. 2b) and crypt depth significantly increased (*P* < 0.05; Fig. 2c). Furthermore, the transfer of piglets to a nursery caused villus atrophy in the proximal and distal (*P* < 0.05) SI from d3 to d5. Villi were significantly wider (at d19: *P* < 0.001) and crypts deeper (at d8 and d19: *P* < 0.01) in artificially reared piglets compared to sow-reared piglets. In the distal SI of NBW sow-reared piglets villus heights were consistently higher compared to those of their LBW littermates (*P* < 0.05). In contrast, villus heights in the distal SI of NBW artificially reared piglets were on average 193 μm lower at d19 compared to their LBW littermates (*P* < 0.05).

### PCNA and caspase-3 protein expression in the proximal and distal small intestine

**Fig. 3:**
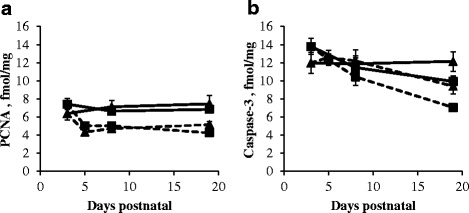
Relative protein expression of PCNA (fmol/mg) (**a**) and caspase-3 (fmol/mg) (**b**) in the proximal (*square*) and distal (*triangle*) SI of sow- (*full line*) and artificially reared (*dashed line*) piglets. Values are means ± SE (*n* = 12 as LBW and NBW piglets were pooled together)

PCNA protein expression remained at the same level during the suckling period in the proximal and distal SI of sow-reared piglets (Fig. 3a). Transferring the piglets at d3 to a nursery significantly reduced PCNA protein expression in the proximal SI at d5 (*P* < 0.05) as compared to d3, whereas in the distal SI its level remained unaffected. PCNA protein expression in the proximal and distal SI of artificially reared piglets was significantly lower compared to sow-reared piglets at d8 (*P* < 0.05) and at d19 in the proximal SI (*P* < 0.001) (Fig. 3a).

Caspase-3 protein expression significantly decreased in the proximal SI of sow-reared (*P* < 0.05) and artificially reared piglets (*P* < 0.001) piglets from d3 to d19 (Fig. 3b). In contrast, in the distal SI of sow- and artificially reared piglets, no age-related differences were observed. Caspase-3 protein expression was significantly lower in the proximal (*P* < 0.05) and distal (*P* < 0.05) SI of artificially reared piglets compared to sow-reared piglets at d19.

### Occludin and claudin-3 protein expression in the proximal and distal small intestine

**Fig. 4:**
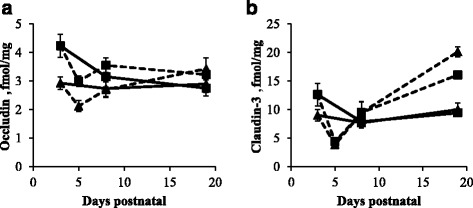
Relative protein expression of occludin (fmol/mg) (**a**) and claudin-3 (fmol/mg) (**b**) in the proximal (*square*) and distal (*triangle*) SI of sow- (*full line*) and artificially reared (*dashed line*) piglets. Values are means ± SE (*n* = 12 as LBW and NBW piglets were pooled together)

Occludin expression in the proximal SI of sow-reared piglets decreased from d3 to d19 (*P* < 0.001), whereas in the distal SI its level remained unchanged (Fig. 4a). Occludin expression dropped significantly in the proximal (*P* < 0.05) but not in the distal SI of artificially reared piglets, from d3 to d5, but returned to its initial levels afterwards. The expression of occludin in the SI was comparable for sow- and artificially reared piglets.

Claudin-3 expression in the proximal and in the distal SI of artificially reared piglets dropped significantly from d3 to d5 (*P* < 0.01; Fig. 4b). Claudin-3 expression increased from d5 to d19 and was significantly higher in the SI of artificially reared piglets compared to sow-reared piglets at d19 (*P* < 0.01). Claudin-3 expression in the distal SI of LBW sow-reared piglets was on average 33% higher compared to NBW sow-reared piglets at d3, d8 and d19 (*P* < 0.01).

### Ex vivo permeability in the proximal and distal small intestine

**Fig. 5:**
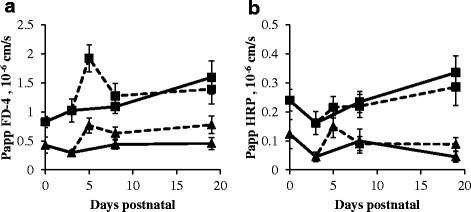
Intestinal permeability indicated by the Papp of FD-4 (10^-6^ cm/s) (**a**) and HRP (10^-6^ cm/s) (**b**) in the proximal (*square*) and distal SI (*triangle*) of sow- (*full line*) and artificially reared (*dashed line*) piglets. Values are means ± SE (*n* = 6)

FD-4 and HRP permeability in the proximal and distal SI remained stable in sow-reared piglets (Fig. 5a and b). FD-4 permeability in the proximal SI significantly increased in 5-day-old NBW artificially reared piglets when transferred to a nursery at d3 (*P* < 0.05; Fig. 5a). Given the observation that the Papp of FD-4 was already high at 3 days of age in the proximal SI of LBW piglets, no changes could be observed after the start of artificial rearing. In the distal SI, both FD-4 (*P* < 0.05; Fig. 5a) and HRP (*P* < 0.01; Fig. 5b) permeabilities were significantly increased in both 5-day-old LBW and NBW artificially reared piglets, compared to d3. LBW sow-reared piglets consistently showed significantly higher FD-4 (proximal: on average 0.5 × 10^-6^ cm/s higher; distal: on average 0.2 × 10^-6^ cm/s higher) and HRP (proximal: on average 0.09 × 10^-6^ cm/s higher; distal: on average 0.06 × 10^-6^ cm/s higher) permeability when compared to their NBW littermates (*P* < 0.05). No such differences were observed in artificially reared piglets.

### Mucosal redox state represented by GSH concentration, GSSG concentration, GSSG/GSH ratio, GSH-Px activity and MDA concentration

**Fig. 6:**
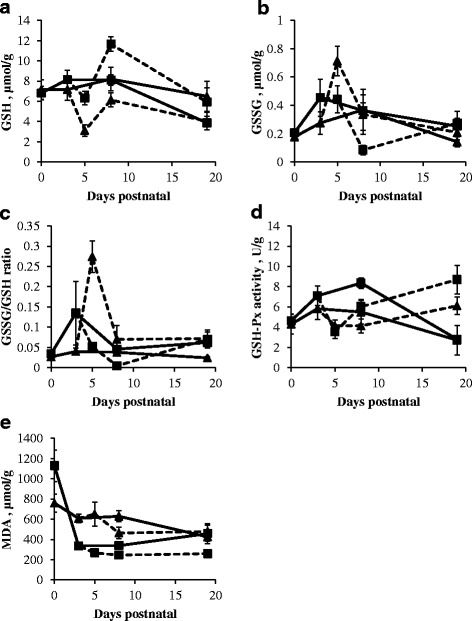
Mucosal GSH concentration (μmol/g) (**a**), GSSG concentration (μmol/g) (**b**), GSSG/GSH ratio (**c**) and GSH-Px activity (U/g) (**d**), MDA concentration (μmol/g) (**e**) of the proximal (*square*) and distal SI (*triangle*) in sow- (*full line*) and artificially reared (*dashed line*) piglets. Values are means ± SE (*n* = 12 as LBW and NBW piglets were pooled together)

GSH concentration was constant from birth until d8. In the distal SI mucosa GSH concentrations remained stable SI, whereas in the proximal SI it decreased significantly after d8 (*P* < 0.01) of sow-reared piglets (Fig. 6a). The concentration of GSSG and the GSSG/GSH ratio did not show any age-related changes in sow-reared piglets (*P* > 0.05; Fig. 6b and c). The activity of GSH-Px in the proximal and distal SI mucosae of sow-reared piglets increased from birth until d3 (*P* < 0.05). After d8, GSH-Px activity decreased significantly (*P* < 0.01), reaching a similar activity at d19 as observed at birth (Fig. 6d).

In the proximal intestine of sow-reared pigs, the concentration of MDA abruptly dropped after birth (*P* < 0.001; Fig. 6e) and remained stable from d3 until d19, whereas in the distal intestine the decrease was more spread out in time (*P* < 0.01). Transferring piglets to a nursery decreased the concentration of GSH, whereas the GSSG concentration and GSSG/GSH ratio were increased in the SI of artificially reared piglets at d5. In the proximal SI of artificially reared piglets GSH concentration peaked, while GSSG concentration and GSSG/GSH ratio showed a minimum at d8 (*P* < 0.05). However by d19, these redox parameters returned to the values noted at birth (*P <* 0.05). GSH-Px activity showed a minimum at d5 (*P* < 0.05), but recovered from d8 to d19 (*P* < 0.01) in the SI of artificially reared piglets.

In the proximal SI, GSH concentration was significantly higher (*P* < 0.05), while GSSG concentration (*P* < 0.01) and consequently the GSSG/GSH ratio (*P* = 0.001) was significantly lower in artificially reared piglets compared to sow-reared piglets at d8. GSH-Px activity was significantly lower at d8 (*P* < 0.001) and significantly higher at d19 (*P* < 0.001) in both regions of the SI of artificially reared piglets compared to sow-reared piglets. GSH-Px activity in the SI of LBW sow-reared piglets was significantly higher than their NBW littermates (proximal: on average 1.38 U/g higher; distal: on average 0.64 U/g higher) (*P* < 0.001). A similar observation for both birth weight categories was made in the distal SI of artificially reared piglets (on average 1.24 higher, *P* < 0.01).

At d8, MDA concentration in the proximal SI of sow-reared piglets was significantly higher compared to artificially reared piglets (*P* < 0.01).

### Liver redox state represented by GSH concentration, GSSG concentration, GSSG/GSH ratio, GSH-Px activity and MDA concentration

**Fig. 7:**
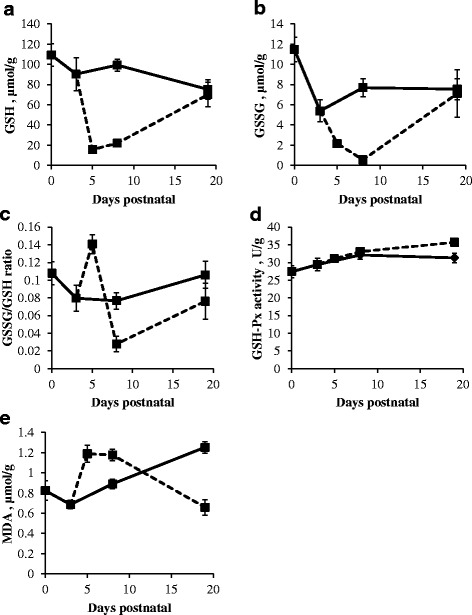
Liver GSH concentration (μmol/g) (**a**) and GSSG concentration (μmol/g) (**b**), GSSG/GSH ratio (**c**), GSH-Px activity (U/g) (**d**) and MDA (μmol/g) (**e**) in sow- (*full line*) and artificially reared (*dashed line*) piglets. Values are means ± SE (*n* = 12 as LBW and NBW piglets were pooled together)

Liver GSH concentration, GSSG/GSH ratio and GSH-Px activity in sow-reared piglets did not significantly change (Fig. 7a, c and d). GSSG concentration decreased significantly from d0 to d3 (*P* < 0.05; Fig. 7b) in sow-reared piglets and returned to concentrations seen at birth afterwards (Fig. 7b). MDA concentration showed a significant increase after d3 in both LBW and NBW piglets (*P* < 0.05; Fig. 7e).

When piglets were introduced to a milk replacer, GSH (*P* < 0.05) and GSSG (*P* < 0.01) concentrations significantly decreased whereas GSSG/GSH ratio (*P* < 0.05) and MDA concentration (*P* < 0.001) significantly increased from d3 to d5. Meanwhile, GSH-Px activity remained unchanged. All redox parameters in the liver were stable from d5 to d8 in artificially reared piglets, except GSSG/GSH ratio that showed a significant decrease (*P* < 0.01). Towards d19, GSH concentration (*P* < 0.01) and GSH-Px activity (*P* < 0.05) significantly increased while MDA concentration (*P* < 0.01) significantly decreased in artificially reared piglets.

At d8, a lower GSH concentration and a higher MDA were observed in artificially reared piglets compared to sow-reared piglets (*P* < 0.001). At d19, higher GSH-Px activities were observed in artificially reared piglets compared to sow-reared piglets (*P* < 0.01).

### Systemic redox state represented by GSH concentration, GSSG concentration, and GSSG/GSH ratio, GSH-Px activity and MDA concentration

**Fig. 8:**
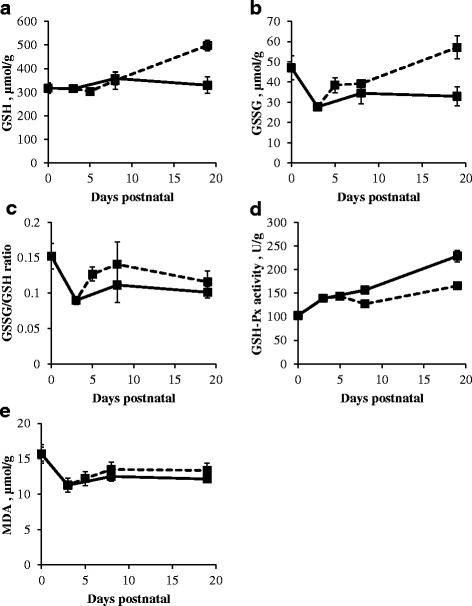
Systemic GSH concentration (μmol/g) (**a**), GSSG concentration (μmol/g) (**b**), GSSG/GSH ratio (**c**), GSH-Px activity (U/g) (**d**) and MDA concentration (μmol/g) (**e**) in sow- (*full line*) and artificially reared (*dashed line*) piglets. Values are means ± SE (*n* = 12 as LBW and NBW piglets were pooled together)

The GSH-Px activity gradually, significantly increased during the investigated time frame in both sow- and artificially reared piglets (*P* < 0.01; Fig. 8d). GSH concentration in erythrocytes significantly increased in artificially reared piglets (*P* < 0.01) from d5 to d19, but remained constant in sow-reared piglets from d0 to d19 (Fig. 8a). Plasma MDA concentration decreased 6.77 μmol/g during the first 3 days of life in LBW piglets (*P* < 0.01; Fig. 8e). Plasma GSH-Px activity was significantly lower in artificially reared piglets compared to sow-reared piglets at d8 and d19 (*P* < 0.05). At d19, GSH-Px activity was still lower when LBW piglets were artificially reared (*P* < 0.05). Erythrocyte GSSG concentrations (*P* < 0.01; Fig. 8b) and GSSG/GSH ratio (*P* < 0.05; Fig. 8c) were significantly increased at d5 when artificially reared piglets were transferred to a nursery at d3 (Fig. 8b and c). The concentration of GSH (*P* < 0.01) and GSSG (*P* < 0.05) significantly increased in artificially reared piglets from d5 to d19. At d19, GSH concentration and GSSG concentration were significantly higher in artificially reared when compared to sow-reared piglets (*P* < 0.05). GSH concentration was significantly lower in LBW sow-reared piglets when compared to their NBW littermates. (*P* < 0.05).

## Discussion

Given the need for an alternative rearing strategy that lowers the challenges that LBW and supernumerary piglets face during the suckling period, we aimed to investigate the responses to full artificial rearing. Our data demonstrated that artificial rearing beneficially affected piglet performance, notwithstanding impairing effects on small intestinal architecture, permeability and redox state in both LBW and NBW piglets.

### Artificial rearing influences piglet performance

This study documents the implications of full artificial rearing of LBW and supernumerary piglets. Under standard rearing conditions these pigs are at high risk of succumbing due to insufficient nutrient intake, increased disease susceptibility, and physiological deficits (e.g. lower energy reserves) [1]. Our study demonstrated that transitioning piglets to a nursery with ad libitum access to a milk replacer led to significantly higher body weights of LBW and NBW piglets compared to sow-reared piglets at d19. The experiment was terminated on the same day as when the conventionally reared piglets were weaned on the farm with a 3-week batch system. For this specific farm, the average age at weaning is 19.6 d. Furthermore, milk production of the sow strongly decreases towards the end of the suckling phase. Around d 18–19, sow milk starts to be very limiting and becomes hard to compare to the ad libitum access for the artificially reared piglets [43]. Using a weigh-suckle-weigh technique, De Vos et al. [26] showed that piglets with ad libitum access to milk replacer have a higher relative energy intake compared to sow-reared piglets [28]. Our findings confirm this and other previous research where LBW piglets receiving an energy rich diet—comparable with our LBW piglets fed a milk replacer ad libitum—presented a comparable body weight gain as NBW piglets receiving a lower energy intake—comparable with our NBW piglets fed by the sow [44]. In addition, the milk replacer used in our study contained spray-dried plasma which could have contributed further to the higher weight gain in the artificially reared group. Ermer et al. [45] showed that spray-dried porcine plasma increased feed intake. Thus next to ad libitum access to feed, diet composition cannot be neglected.

### Artificial rearing influences small intestinal architecture

Small intestinal morphology is one of the major indicators reflecting gut health in pigs [46]. However, caution should be taken when evaluating morphology alone as a measure of gut health. For example, Enterotoxigenic *Escherichia coli*, the major causal agent of neonatal diarrhea, may occur without histological changes in the intestine [47]. Notwithstanding, stereological analysis of small intestinal morphology will provide the most accurate estimation of the intestinal absorptive surface area [48], a proxy of the surface can be calculated using villus height and villus width [47]. In our study, the mucosal surface area of the proximal small intestine at d19 was markedly larger in artificially reared piglets than sow-reared piglets. In this regard, feeding a milk replacer shows promise as the increased mucosal surface suggests a higher ability to absorb nutrients. Moreover, previous research showed an increased activity of maltase and sucrase when piglets are fed a milk replacer [49–51]. Thus, artificial rearing seems to improve the digestive capacity at the level of the small intestine.

The deepening of the crypts could be a response to promote mucus secretion rather than lead to enterocyte maturation and proliferation and thus an increase in PCNA expression. Previous research showed that breast-fed infants showed a delay in the mucin degradation when compared to artificially reared infants [52]. Phillips [53] showed that crypt goblet cells have the ability to restitute the mucus layer and showed a decrease in the percentage of villus epithelial volume occupied by mucin secretory granules.

Transferring piglets to a nursery at d3 exposed them to stressful effects caused by psychological, environmental or nutritional factors similar to those encountered during the conventional weaning process [54]. However, it is difficult to unravel the separate contributions of these factors. Previous studies showed significant villus atrophy at d4 and deeper crypts at d7 in piglets that were separated from the sow and still fed sow’s milk compared to unweaned piglets [55, 56]. Similarly, our study showed transient villus atrophy in piglets transferred to a nursery at d3. This villus height reduction is analogue to the intestinal morphological changes as a result of inadequate food intake immediately after conventional weaning [56–59]. In contrast to conventional weaning, villus length is rapidly restored. This could be related to the inclusion of spray-dried plasma in the milk replacer since this is known to increase villus height [8, 60–63].

### Artificial rearing affects small intestinal tight junction protein expression

We hypothesized that artificial rearing influences small intestinal physiology. The intestinal epithelium plays a critical role in the transport of nutrients and macromolecules. At the same time, it has to provide an effective barrier to harmful macromolecules and microorganisms [64]. Epithelial cells constitute a dynamic barrier where large molecules can be transported by transcytosis and this can be measured by HRP [65]. Tight junctions (TJs) are essential components of the physical intercellular barrier and their presence and functionality changes under different physiological and pathological conditions [66, 67]. Well-formed TJs are characterized by low solute permeability which can be determined by measuring FD-4 permeability [65]. The family of junctional adhesion molecules, the claudin and occludin families, are structural transmembrane TJ components that have the potential to mediate cell–cell adhesion [66, 68]. Within TJs, claudins are the main determinants of the selective pore properties [68, 69], while the role of occludin in barrier functioning is more diverse [70, 71]. In mice, the expression of claudin-3 is promoted during the first 3 weeks of life concomitant with the establishment of the intestinal microbiota [72, 73]. Our study showed that claudin-3 expression rose particularly in artificially reared piglets. Possibly the different microbiota fingerprint [74] and the absence of milk born IgA’s [34] can be held responsible. Claudin-3 is known to be a “tightening” claudin [75]. This could explain why permeability is seemingly unaffected by the observed increase in claudin-3. However, at the start of artificial rearing, claudin-3 as well as occludin protein levels transiently dropped. This drop is reflected in a concomitantly increased permeability for FD-4 and HRP. Previous studies showed lower abundances of occludin mRNA and protein, claudin-3 protein, and increased lactulose permeability after weaning [50, 76]. Thus artificial rearing induced a similar response as seen after conventional weaning.

### Artificial rearing resulted in a redox imbalance

Previously, we investigated the link between oxidative stress, intestinal integrity, and permeability in intestinal epithelial cells in vitro [12] and in vivo during normal suckling [21]. Vergauwen et al. [12] and others showed a redistribution of TJ proteins during times of imposing reactive species and could relate these responses to a compromised permeability [12, 77]. The current study demonstrated that transfer of piglets to a nursery resulted in oxidative stress.

Glutathione (GSH) is an important regulator of the redox status within intestinal epithelial cells [13]. The liver is the major site of GSH biosynthesis and exports GSH via the bile to the proximal SI [78]. Thus GSH originating from the liver supports mucosal GSH by decreasing lipid peroxidation and maintaining the GSSG/GSH redox homeostasis in the proximal intestine [11, 79, 80]. In our study, artificial rearing resulted in the liver in a decreased GSH content, an increased GSSG/GSH ratio and MDA concentration. After this first phase, the redox parameters returned to their initial values. GSH-Px activity in the liver seemed unaffected during the transition period, while GSH-Px activity transiently dropped in the proximal and distal SI when piglets were introduced to a nursery. Previously, conventional weaning caused a drop in GSH-Px activity but GSH-Px activity increased afterwards as part of a feedback mechanism [31]. Our study shows that artificial rearing increases oxidative stress in the SI and as a result the GSH-Px activity increased and was higher than the activity seen in sow-reared piglets. Furthermore, LBW piglets showed a higher GSH-Px activity compared to their NBW littermates. It is clear that the antioxidant capacity of the liver helps protecting the proximal intestine by secreting GSH into the lumen of proximal SI [78, 79]. This could explain the increased mucosal GSH concentration of the proximal SI and the concomitant massive decrease of the liver GSH concentration. On the other hand, a remarkable drop of the GSH concentration in the distal SI was observed from d3 until d5. Consequently, this resulted in a high concentration of MDA and GSSG, resulting in a higher GSSG/GSH ratio in the distal SI. Furthermore, the GSSG/GSH ratio increased systemically upon transfer to a nursery. Degroote et al. [31] already showed that conventional weaning increased the GSSG/GSH ratio.

Our research presents a window of opportunity for antioxidant supplementation to protect piglets from redox imbalance due to artificial rearing. NBW artificially reared piglets were more susceptible to redox imbalance and loss of intestinal integrity upon transferal to a nursery (from d3 to d5). Taken together, these findings favor the transfer of both LBW and NBW piglets to a nursery as a solution for LBW and supernumerary piglets. Further research is necessary to elucidate how these artificially reared LBW and NBW piglets will respond to the introduction to solid food.

## Conclusions

In conclusion, we demonstrated that artificial rearing influences morphological and functional parameters in the small intestine, liver and blood in a way similar to what is seen after conventional weaning. Nevertheless, growth performance of artificially reared piglets was positively influenced. In addition, artificially reared piglets rapidly recovered from redox imbalances and restored intestinal permeability within a couple of days. Further research is needed to explore the possibility to supplement LBW and NBW piglets with antioxidants prior to initiating artificial rearing. Thus, artificial rearing is a valuable alternative to raise LBW or supernumerary piglets.

## Abbreviations

ELISA: Enzyme linked immunosorbent assay
FD-4: Fluorescein isothiocyanate dextran 4 kDa
GSH: Glutathione
GSH-Px: Glutathione peroxidase
GSSG: Oxidized glutathione
HPLC: High performance liquid chromatography
HRP: Horseradish peroxidase
LBW: Low birth weight
MDA: Malondialdehyde
NBW: Normal birth weight
SI: Small intestine
TJs: Tight junctions
